# Various Antihypertensive Drug Compliance Models and Cardiovascular Prognosis of People With Incidentally Detected High Blood Pressure During Health Check‐Up in Korea

**DOI:** 10.1155/ijhy/5581168

**Published:** 2025-12-30

**Authors:** Jong-Kwan Park, Sungyoun Chun, Joongmin Kim, Hancheol Lee, Ji-Yong Jang, Kyeong-Hyeon Chun, Hyeongsoo Kim, Seung-Jin Oh, Geunhee Park, Se-Jung Yoon

**Affiliations:** ^1^ Division of Cardiology, National Health Insurance Service Ilsan Hospital, Goyang, Republic of Korea, nhimc.or.kr; ^2^ Research Institute National Health Insurance Service Ilsan Hospital, Goyang, Republic of Korea

**Keywords:** cardiovascular disease, health check-up, hypertension, medication

## Abstract

**Background:**

A health check‐up system (HCS) is one of the best ways to prevent complications and maintain health by diagnosing diseases and screening risk factors early. Here, we investigated how many people who detected elevated blood pressure through the HCS were finally diagnosed with “hypertension” and continuously treated. We also analyzed their cardiovascular risk and prognostic significance according to the multiple drug compliance patterns.

**Methods:**

A total of 38,100 subjects without cardiovascular disease who were newly detected with elevated blood pressure in HCS between 2006 and 2011 were analyzed and followed up until 2019 using the Korean National Health Insurance Database. They were divided into five subgroups through subsequent prescription history and compared for epidemiological, laboratory performance and cardiovascular events.

**Results:**

Of the total 38,100 subjects, 6981 (18.3%) cases were diagnosed with hypertension and started medication within 12 months. Of those cases, 3021 (7.9%) cases continued taking their medication, 2184 (5.7%) cases persistently discontinued medication, and 485 (1.3%) cases restarted medication again within 12 months of discontinuation. As a result of follow‐up until 2019, the “drug‐free group” showed the significantly lowest cardiovascular complication incidences (angina, heart failure, ischemic heart disease, CKD, and PAOD), and the highest were seen in the “re‐initiation group” (cerebral infarct and atrial fibrillation) compared with the “continuous medication group.”

**Conclusions:**

A considerable proportion of individuals with high blood pressure detected in HCS were diagnosed with hypertension and at high cardiovascular risk. The group that needed to restart medication within 12 months after discontinuation showed a higher cardiovascular risk among them.

## 1. Introduction

High blood pressure is the most important modifiable risk factor and is also the biggest contributor to cardiovascular and cerebrovascular diseases [1–3]. In the twenty‐first century, cardiovascular and cerebrovascular disease‐related mortality accounts for nearly half of all deaths in the developed regions and for one‐quarter in the developing regions [4]. Hypertension has a high prevalence, affecting approximately 30% of the adult population worldwide, with a similar prevalence in Korea. The number of patients with hypertension has steadily increased to more than 12 million in South Korea (hereafter referred to as Korea) [2, 3, 5]. Mortality rates from cardiovascular diseases are declining in Korea; nonetheless, heart disease, cerebrovascular disease, and hypertension remain the second, fourth, and tenth leading causes of death, respectively [6].

A health check‐up system (HCS) is one of the best ways to prevent diseases and maintain health by screening for risk factors and diagnosing diseases early. As the burden of illness shifts from infectious to chronic diseases, the importance of health check‐up is increasingly emphasized. Korean health authorities began the National Health Screening Programs (NHSPs) for public servants and private school staff in 1980. The general health check‐up included lifestyle questionnaires, anthropometric measurements, blood pressure measurement, visual acuity test, hearing test, and laboratory tests including fasting glucose, lipid profile, liver function tests, creatinine, urinalysis, and chest radiography. Additionally, bone density, cognitive function, and depression were assessed. Testing for noncommunicable diseases has improved the control rates of chronic diseases, such as hypertension and diabetes mellitus. To discover and treat disease early and improve health, the National Health Insurance Service (NHIS) provides regular health check‐ups and a cancer screening program. All insured Koreans who are at least 40 years old and their dependents can receive biannual health checks without cost [7–9].

In particular, there are many people who are recommended to visit a hospital after health check‐up and start detailed examinations or treatments for abnormal findings they had not known before. Favorable changes in control of cardio‐cerebrovascular risk factors may lead to a decline in age‐standardized mortality and heart disease over several decades [7–9]. In fact, in case of hypertension or diabetes, which occurs without symptoms and progresses, there are many people who are diagnosed through HCS incidentally, being introduced into a continuous treatment process later.

Here, we investigated how many people who detected elevated blood pressure through the health screening system were finally diagnosed with “hypertension” and continuously treated. Furthermore, we analyzed whether they actually had a significant cardiovascular risk and aimed to evaluate the clinical and prognostic significance of the high blood pressure detected through HCS.

## 2. Materials and Methods

### 2.1. Statement of Ethics

This study adhered to the tenets of the Declaration of Helsinki, and the NHIS‐National Sample Cohort 2.0 database (NHIS‐NSC) 2006–2019 project of the National Health Insurance Database (NHID) was approved by the Institutional Review Board of the Korean National Health Insurance Service (KNHIS). This study design was reviewed and approved by the Institutional Review Board of the NHIS Ilsan Hospital, Gyeonggi‐do, Korea (NHIMC 2021‐03‐027). As personal information was strictly masked during cohort generation according to the guidelines of the Korean Health Insurance Review and Assessment Service, written informed consent was waived.

### 2.2. Data Source and Study Population

Korea implements a single‐payer, universal, mostly fee‐for‐service, and compulsory healthcare insurance system. Korea’s health insurance system started to be implemented for workplaces with 500 or more workers in 1977, and it has been expanded to nationwide medical insurance since 1989. All Koreans residing in Korean territories are covered under one of the following three categories: employee insured, self‐employed insured, and medical aid beneficiary [8–11]. The NHI program is a wage‐based, contributory insurance program covering about 97% of the population, while the Medical Aid program is a government‐subsidized public assistance program for those of low income and medically indigent individuals [12–15].

So, nearly all of the data in the health system are centralized in large databases. In Korea, patients with KNHIS pay approximately 30% of their total medical expenses when using medical facilities, and medical providers are required to submit claims for the remaining 70% of the medical expenses. Claims are accompanied by data regarding diagnostic codes, procedures, prescription drugs, personal information about the patient, information about the hospital, the direct medical costs of both inpatient and outpatient care, and dental services. No healthcare records of the patients were duplicated or omitted because all Korean residents have received a unique identification number at birth. This number is used by the Korean government for purposes related to the healthcare system, etc. Further, the KNHIS uses the Korean Classification of Diseases (KCD), which is a similar system to the International Classification of Diseases (ICD) [8–11, 14, 16–21].

This study was a nationwide, noninterventional, retrospective registry study using claims database already available in electronic patient records from KNHIS for the application of medical insurance. For this study, we used National Health Insurance Cohort (NHI Cohort) data (2006–2019). The NHI cohort consists of sampling data from about 1 million people, 2.2% of the total population of Korea. The data provided by the NHI cohort included medical history details, national health examination results, information on health insurance qualifications and medical institutions, and birth and death information of all Korean National Health Insurance and medical aid beneficiaries from 2006 to 2019. The subjects’ data were anonymized [12, 22]. This study used NHIS‐NSC data (NHIS‐2021‐2‐007), made by the NHIS. We have enrolled the cases without any cardiovascular disease including hypertension in which high blood pressure was newly detected on HCS in the sample cohort between 2006 and 2011. Previously diagnosed hypertension and any cardiovascular disease were excluded. They were analyzed and followed up until 2019.

### 2.3. Definition of High Blood Pressure, ICD Code for Hypertension, Classification of Subgroups, and Definition of Cardiovascular Disease

Subjects with no previous medical claims for hypertension and any cardiovascular disease with newly detected high blood pressure (systolic blood pressure of 140 mmHg or more or diastolic blood pressure of 90 mmHg or more) on HCS were enrolled [23]. The ICD code for hypertension was expressed as “I10.” They were divided into five subgroups through subsequent prescription history and compared for epidemiological, laboratory performance and cardiovascular events.

Subgroups are defined as follows: “drug‐free group” refers to cases where there has been no medical record for the diagnosis of “hypertension” and medication during the observation period as high blood pressure was found on HCS. The “continuous medication group” refers to cases where high blood pressure is detected on HCS and treatment for hypertension begins within 12 months and continues.

In this study, “discontinuation of treatment” was defined when there was no claim for medical expense of prescription of antihypertensive medication for a period exceeding 6 months within 12 months of starting drug treatment. The “no re‐initiation group” refers to cases that started antihypertensive medication within 12 months after high blood pressure was detected in the HCS, but discontinued it within 12 months of initiation and remained off medication throughout the observation period. The “re‐initiation group” refers to cases where hypertension was detected on HCS, and treatment was started within 12 months, but was discontinued within 12 months from starting medication and then resumed medication within 12 months for some reason. The group of “the others” was classified as various cases where there were medical records of medication or diagnosis of “hypertension” at least once during the observation period, but starting or stopping medication after 12 months or taking medication irregularly.

All subjects were followed for cardiovascular events, such as acute myocardial infarction (AMI), angina, cerebral infarct, atrial fibrillation, ischemic heart disease, stroke, chronic kidney disease (CKD), peripheral artery occlusive disease (PAOD), and heart failure by searching KCD codes.

### 2.4. Statistical Analysis

For statistical analysis, the chi‐square test was used to compare different characteristics of categorical variables of demographic and cardiovascular event information, and one‐way analysis of variance (ANOVA) was used to determine the differences of mean values in demographic and health check‐up results of the participants with elevated blood pressure in HCS. We performed post hoc analyses to examine differences in demographic data, laboratory data, and event incidence among the five subgroups. Continuous variables were compared using one‐way ANOVA, followed by Tukey’s honestly significant difference (HSD) test for pairwise comparisons. Categorical variables were analyzed using the chi‐square test, and post hoc pairwise comparisons were conducted with Bonferroni‐adjusted *p*‐values. All tests were two‐sided, and a *p*‐value of less than 0.05 was considered to indicate statistical significance.

We observed the incidence of the adverse cardiovascular events according to the subsequent drug compliance pattern after health check‐up during the observation period and plotted the results using the Kaplan–Meier method. Additionally, the Cox proportional hazards model was used to determine the risk of complications according to participation in subgroups. Results of the survival analysis were expressed as hazard ratio (HR) and their 95% confidence interval (CI). All covariates were adjusted, and calculated *p*‐values were two‐sided and assumed significant at *p* < 0.05. All results were analyzed using SAS 9 (SAS Institute, Cary, NC, USA).

## 3. Results

### 3.1. Demographic Data of Subjects With High Blood Pressure on HCS

A total of 38,100 subjects with no cardiovascular disease including hypertension but high blood pressure on health check‐up were analyzed. The median duration of follow‐up was 9.78 years (3571 days).

**Figure 1:**
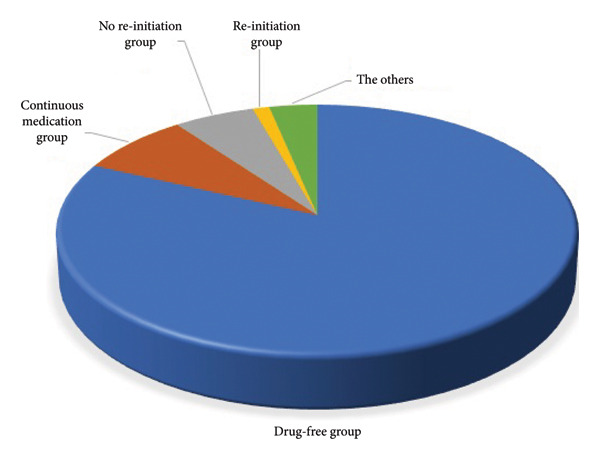
Subgroups with high blood pressure detected in health check‐up of the total 38,100 subjects, 6981 (18.3%) cases were diagnosed with hypertension and started medication within 12 months. Of those cases, 3021 (7.9%) cases continued to take the medicine, 2184 (5.7%) cases continuously discontinued medication, and 485 (1.3%) cases resumed medication again within 12 months. The others (1,049) accounted for 3.3%.

Of the total 38,100 subjects, 31,119 (81.7%) cases did not take any antihypertensive drug and were not diagnosed with hypertension during the follow‐up period, and 6981 (18.3%) cases were diagnosed with “hypertension” and started medication within 12 months. The drug compliance after diagnosis of hypertension varied widely. Of those cases, 3021 (7.9%) cases continued to take the medicine during the observational period. In 2184 (5.7%) cases, medication was discontinued within 12 months of initiation and continued during the observation period. In addition, there were 485 (1.3%) cases in which medication was resumed within 12 months after discontinuation and continued. The other group accounted for 3.4% (1291) (Figure 1).

Among the groups in which high blood pressure was first discovered, 10,015 (26.3%) were under the age of 40, 11,055 (29.0%) were in their 40 s, 9578 (25.1%) were in their 50 s, 5258 (13.8%) were in their 60 s, and 2194 (5.8%) were over the age of 70. There were more males overall (26,601 [69.8%] vs 11,499 [30.2%]), and diabetes and dyslipidemia were 1758 (4.6%) and 1837 (4.8%), respectively. People under the age of 50 were the most common (59.2%) in the “drug‐free group,” and those in their 50 s were the most common (36.8%) in the other four subgroups. Body mass index (BMI) was not significantly different among subgroups (Table 1).

**Table 1:** Demographic data of subgroups of high blood pressure on health screening without cardiovascular disease.

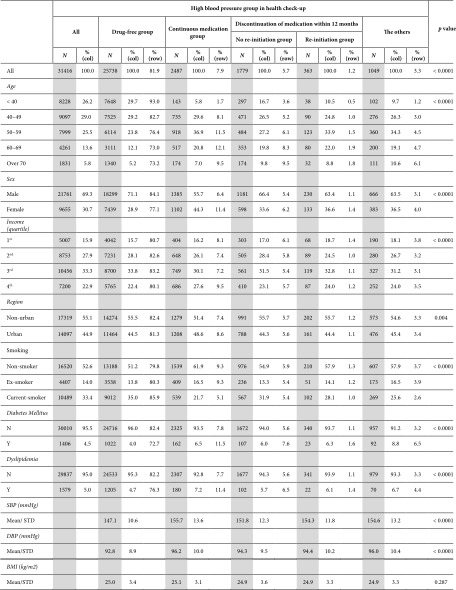

*Note:* Urban, city with a population of 1 million or more; income, divided into 4 equal parts based on insurance payment (1st quartile; the lowest group).

Abbreviations: BMI, body mass index; DBP, diastolic blood pressure; SBP, systolic blood pressure.

With post hoc analysis, the “drug‐free group” showed a significantly higher male proportion and lower systolic and diastolic blood pressure levels compared to the other four subgroups diagnosed with hypertension. In addition, the “drug‐free group” presented a significantly lower diabetes rate compared to the “continuous medication group,” “no re‐initiation group,” and “the other group,” and it also showed a significantly lower dyslipidemia rate compared to the “continuous medication group” and “the other group” (Supporting information 1).

### 3.2. Laboratory Data of Subjects With High Blood Pressure on HCS

Total cholesterol levels were the highest in the “continuous medication group” (208.0 ± 41.7 mg/dL) and the “re‐initiation group” (208.2 ± 39.1 mg/dL) compared with the “drug‐free group” (202.8 ± 39.2 mg/dL). Fasting blood sugar levels were the highest in the “re‐initiation group” (105.6 ± 38.6 vs 100.0 ± 26.3 mg/dL, data of the “drug‐free group”) (Table 2).

**Table 2:** Laboratory data of subgroups of high blood pressure on health screening without cardiovascular disease.

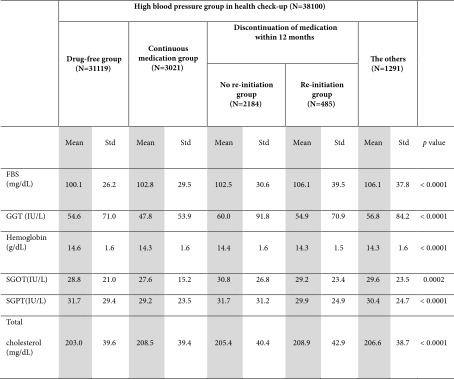

*Note:* SGOT, aspartate aminotransferase; SGPT, alanine aminotransferase.

Abbreviations: FBS, fasting blood sugar; GGT, gamma‐glutamyl transferase.

The “drug‐free group” had significantly lower fasting blood glucose level compared to the other four subgroups with a post hoc analysis. It also showed significantly lower total cholesterol level compared to the “continuous medication group” and “re‐initiation group” in a post hoc analysis (Supporting Table 2).

### 3.3. The Incidence of Cardiovascular Events in Subjects With High Blood Pressure on HCS

Among patients found to have high blood pressure during HCS, 18.3% were eventually diagnosed with hypertension and started to take medication, and their cardiovascular disease risk was also high. Among the total of 38,100 subjects, the incidence of cardiovascular events was angina (3458/9.1%), atrial fibrillation (695/1.8%), AMI (519/1.4%), stroke (2610/6.9%), heart failure (2100/5.5%), ischemic heart disease (4348/11.4%), CKD (496/1.3%), and PAOD (5457/14.3%), respectively.

The group of “re‐initiation group” showed the highest levels of incidence rate of all cardiovascular events, and “no re‐initiation group” also revealed a higher incidence rate of atrial fibrillation, AMI, and cerebral infarct. The “continuous medication group” showed higher incidence rate of angina, heart failure, ischemic heart disease, PAOD, and CKD (Table 3).

**Table 3:** The incidence of cardiovascular events in subgroups of high blood pressure on health examination without cardiovascular disease.

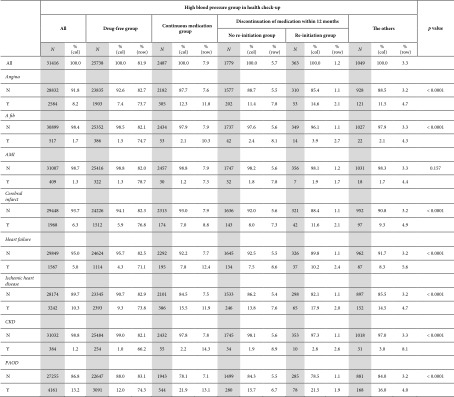

*Note:* A fib, atrial fibrillation.

Abbreviations: AMI, acute myocardial infarction; CKD, chronic kidney disease; PAOD, peripheral arterial occlusive disease.

With post hoc analysis, the “drug‐free group” presented significantly lower rates of most of the cardiovascular events compared to the other four subgroups except for AMI. It revealed significantly lower rates of angina, heart failure, ischemic heart disease, CKD, and PAOD compared to the other four subgroups. The incidence of atrial fibrillation was significantly lower in the “drug‐free group” compared to the “no re‐initiation group” and “re‐initiation group,” and the incidence of stroke was significantly lower in the “drug‐free group” compared to the “no re‐initiation group,” “re‐initiation group,” and “the other group” (Supporting Table 3).

### 3.4. Kaplan–Meier Survival Curve for Cardiovascular Event in Subgroups

**Figure 2:**
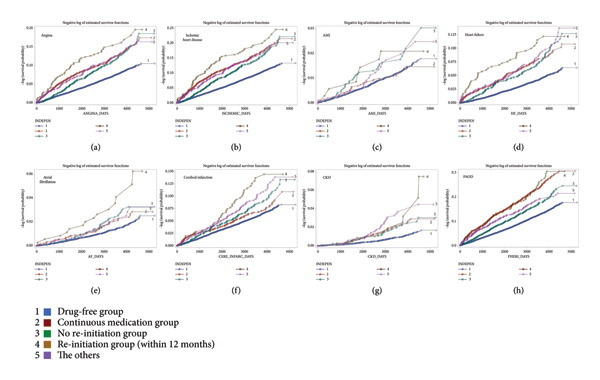
Cardiovascular event rate in the subgroups using the Kaplan–Meier curve. The Kaplan–Meier curve for adverse cardiovascular events according to subsequent drug compliance patterns after health check‐up. Solid lines indicate the incidence rate of adverse cardiovascular events in each subgroup, respectively. Line of blue (drug‐free group), red purple (continuous medication group), green (discontinuation within 12 months and sustained discontinuation group), pale brown (discontinuation within 12 months and re‐initiation within 12 months), and light purple (the others) indicate each pattern of drug compliance. (a) Angina; (b) ischemic heart disease; (c) acute myocardial infarction; (d) heart failure; (e) atrial fibrillation; (f) cerebral infarction; (g) chronic kidney disease; (h) peripheral arterial occlusive disease. AMI, acute myocardial infarction; CKD, chronic kidney disease; PAOD, peripheral arterial occlusive disease.

In all cardiovascular events, the “drug‐free group” showed the lowest complication incidences, and the highest complication incidences were seen in the group of “re‐initiation group.” The “continuous medication group” and the group of “no re‐initiation group” revealed moderate rate of complication incidences (Figure 2).

### 3.5. HR of Cardiovascular Event in Subgroups

As a result of survival analysis based on the group that “continuously took medication for hypertension,” cerebral infarct (HR 1.73, CI 1.23–2.42) and atrial fibrillation (HR 1.84, CI 1.02–3.32) were significantly increased in the “re‐initiation group” when the factors of age, sex, systolic and diastolic blood pressure, smoking, BMI, diabetes, dyslipidemia, and region were adjusted (*p* < 0.05).

Based on the “continuous medication group,” the “drug‐free group” revealed a significantly lower incidence of events, such as angina (HR 0.77, CI 0.69–0.88), HF (HR 0.81, CI 0.69–0.94), ischemic heart disease (HR 0.76, CI 0.68–0.85), CKD (HR 0.64, CI 0.47–0.86), and PAOD (HR 0.71, CI 0.64–0.78) (*p* < 0.01). The “no re‐initiation group” had a significantly higher incidence of cerebral infarction (HR 1.26, CI 1.01–1.58, *p* < 0.05) (Table 4).

**Table 4:** Hazard ratio of cardiovascular disease in each subgroup.

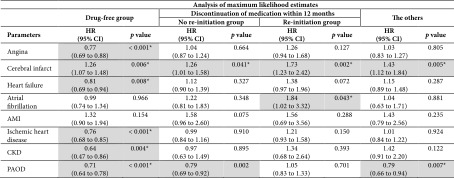

*Note:* A 95% CI for absolute risk reduction attributed to each drug compliance pattern was calculated. Cox proportional hazards models were used to obtain adjusted hazard ratio (HR), 95% CI, and *p*‐values by defining “continuous medication group” as the reference arm. Parameter estimates: age, sex, systolic and diastolic blood pressure, smoking, BMI, diabetes, dyslipidemia, region, and income status. Bolded areas in the table are statistically significant.

Abbreviations: AMI, acute myocardial infarction; CKD, chronic kidney disease; PAOD, peripheral arterial occlusive disease.

^∗^ *p*‐value < 0.05.

## 4. Discussion

This study is the first large‐scale cohort study to show the various antihypertensive drug compliance models after detection of high blood pressure on HCS and to analyze the actual incidence of cardiovascular diseases in several subgroups. Among patients found to have high blood pressure during HCS, a considerable proportion of individuals (18.3%) were eventually diagnosed with “hypertension” and started to take medication, and their cardiovascular disease risk was also high.

The antihypertensive drug compliance patterns after diagnosis of hypertension varied widely. We divided the subjects into five subgroups based on their pattern of antihypertensive medication continuation and compared the characteristics and outcomes of each group. The “drug‐free group” showed the lowest complication incidences, and the highest complication incidences were seen in the “re‐initiation group; medication again within 12 months after discontinuation” in all cardiovascular events by the Kaplan–Meier survival curve. The “continuous medication group” and the group of “no re‐initiation group; consistently discontinued medication” revealed moderate degree of complication incidence in this analysis.

The key point in this study is that a considerable proportion of individuals (18.3%) with elevated blood pressure in the health check‐up were diagnosed with hypertension, which gives the message to concern about elevated blood pressure on health screening. In addition, the “drug‐free group” showed a significantly lower cardiovascular event risk except AMI, atrial fibrillation, and stroke than the “continuous medication group.”

Moreover, the most interesting finding in this study is that the highest cardiovascular events occurred in the group of “re‐initiation group” in the Kaplan–Meier curve and the Cox proportional hazards regression analysis. It revealed the highest risk of atrial fibrillation (HR 1.84, 95% CI 1.02–3.32) and cerebral infarction (HR 1.73, 95% CI 1.23–2.42) significantly. Considering the causal relationship between atrial fibrillation and stroke, the highest probability of occurrence of both of these events with causal relationships strengthened the reliability of the results.

The fact that this group revealed the highest cardiovascular risk indicates that it has been accurately diagnosed with “hypertension” in advance and medication was started. It is presumed that they visited hospitals again after stopping taking drugs due to uncomfortable conditions, such as sustained high blood pressure or various cardiovascular diseases. High accessibility to hospitals or increased vigilance against high blood pressure and cardiovascular disease in Korea would also have played a major role in having patients back to the hospitals after health check‐up. This can be understood in the context of “reverse causality,” as they may have restarted medication due to the occurrence of certain cardiovascular event, as well as sustained high blood pressure. Being in the “re‐initiation of medication group” can be a marker of a pre‐existing high‐risk state, rather than the medication pattern itself being the causal risk factor. From this, it can be seen that the HCS in adults plays an important role in early and accurate detection of these high‐risk vulnerable subjects before the development of symptoms or cardiovascular complications and can provide adequate attention and treatment opportunities for hypertension [20, 21].

Not all people who showed elevated blood pressure at the time of health check‐up were diagnosed with hypertension, and it is assumed that the blood pressure could be temporarily elevated in many cases, or some of them required medication but did not take it. However, regular biennial HCS, high hospital accessibility, overflowing publicity, and education on health in Korea suggest that people are more likely to visit hospitals after detection of high blood pressure. Therefore, a large number of cases who showed high blood pressure at the health check‐up but did not take antihypertensive drugs afterward were likely to be those who showed temporary high blood pressure at screening and were not diagnosed with hypertension by a doctor during the observation period in this study.

Higher levels of blood pressure have been associated with increased cardiovascular risk [24, 25]. In 2010, high blood pressure was the leading cause of death and disability‐adjusted life years worldwide [26, 27]. In the United States, hypertension accounted for more cardiovascular mortality than any other modifiable cardiovascular risk factor and was second only to cigarette smoking as a preventable cause of death for any reason [28]. In a follow‐up study of 23,272 U.S. NHANES (National Health and Nutrition Examination Survey) participants, > 50% of deaths from coronary heart disease (CHD) and stroke occurred among individuals with hypertension [29]. Because of the high prevalence of hypertension and its associated increased risk of CHD, stroke, and end‐stage renal disease (ESRD), the estimated risk of population of these outcomes associated with hypertension is high [29–31].

In Korea, health check‐ups are conducted every two years for adults over the age of 40, and lifestyle adjustment or hospital treatment is recommended for abnormal findings [7, 9, 10]. There are many people who are recommended to consult a doctor for an abnormal finding that was discovered at this health screening and start a detailed examination or treatment. In these cases, it is of great significance that it can be a lucky opportunity to prevent various kinds of complications or mortality that may occur in the future. In fact, in the case of hypertension or diabetes, which occurs without symptoms and progresses, there are many cases that are diagnosed through health check‐up and introduced into a continuous treatment process later.

In summary, among those with elevated blood pressure detected through HCS, a considerable proportion of individuals (18.3%) were finally diagnosed with true hypertension and at high cardiovascular risk. The group that needed to restart medication within 12 months after discontinuation revealed a higher cardiovascular risk among them.

HCS can help in the early detection of hypertension and other metabolic disorders and plays an important role in the early screening of people at high risk for cardiovascular disease.

### 4.1. Study Limitations

The most important limitation is that the diagnosis of several diseases was defined on the basis of KCD codes, which may be inaccurate compared with the diagnoses obtained from a medical chart, and underreporting or misclassification can also be possible. In Korea, accurate diagnostic codes on medical records are essential for medical insurance claims; however, omission, underreporting, or misclassification of medical diagnosis can be possible, and it should be recognized in analyzing the data. Second, because not all subjects receive regular health screening, the NHID may include data based on only parts of the population. Third, the NHID contains only basic laboratory test results; thus, more detailed laboratory results cannot be analyzed. Another limitation is that we did not analyze the type and duration of medication, and it is difficult to know the status of current blood pressure control or the proportion of patients who achieved target blood pressure. In addition, the fact that the detailed reason for stopping medication, such as poor medication compliance, drug side effects, sustained normalized blood pressure, poor accessibility to hospital, or suggestion of doctor, is not known is a very big limitation.

There may be a statistical limitation due to the fact that the “drug‐free group” differs greatly numerically from the other groups. And “the other group” is not easy to identify the nature of heterogeneous characteristics including irregular drug taking. Finally, the exact causes remain unclear for the higher risk of stroke in all groups and the lower risk of PAOD in the drug‐free group, continuously discontinued medication group, and the other groups compared with the continuous medication group with Cox proportional hazards models. More clinical information and additional information of the incidence of diabetes and dyslipidemia during the follow‐up period, as well as more precise prognostic data, would enable better analysis in future studies.

## 5. Conclusions

Early diagnosis of asymptomatic hypertension is very important in reducing the risk of cardiovascular disease. A considerable proportion of individuals with high blood pressure detected in HCS were finally diagnosed as true hypertension and at high cardiovascular risk. Cardiovascular risk analysis according to the various antihypertensive drug compliance patterns revealed different risk levels in large sample data in Korea. HCS can help the early detection of hypertension as well as other metabolic disorders and play a major role in early screening of groups with high cardiovascular risk.

## Ethics Statement

This study design was reviewed and approved by the Institutional Review Board of the NHIS Ilsan Hospital, Gyeonggi‐do, Korea (NHIMC 2021‐03–027). As personal information was strictly masked during cohort generation according to the guidelines of the Korean Health Insurance Review and Assessment Service, written informed consent was waived.

## Disclosure

All the authors critically reviewed and approved the final manuscript.

## Conflicts of Interest

The authors declare no conflicts of interest.

## Author Contributions

Jong‐Kwan Park: study conceptualization, methodology, data curation, and original draft writing. Sungyoun Chun: statistical analysis and review. Joongmin Kim: data curation, review and editing, and visualization. Hancheol Lee: supervision and methodology. Ji‐Yong Jang: methodology and data curation. Kyeong‐Hyeon Chun: conceptualization, and review and editing. Hyeongsoo Kim: methodology, data curation, and writing–editing. Seung‐Jin Oh: conceptualization, supervision, validation, and writing–review and editing. Geunhee Park: review and editing. Se‐Jung Yoon: conceptualization, supervision, methodology, validation, and writing–review and editing.

## Funding

This work was supported by the NHIS Ilsan Hospital Grant (NHIMC 2021‐03–027).

## Supporting Information

Supporting Table 1: Post hoc Analysis‐Demographic data of subgroups of high blood pressure on health screening without cardiovascular disease.

Supporting Table 2: Post hoc Analysis‐Laboratory data of subgroups of high blood pressure on health screening without cardiovascular disease.

Supporting Table 3: Post hoc Analysis‐The incidence of cardiovascular events in subgroups of high blood pressure on health examination without cardiovascular disease.

## Supporting information


**Supporting Information** Additional supporting information can be found online in the Supporting Information section.

## Data Availability

Datasets supporting the findings of this article cannot be shared openly due to the data management policy of the NHIS of Korea but are available from the corresponding author upon reasonable request.
