# Supplementary material for: Various Antihypertensive Drug Compliance Models and Cardiovascular Prognosis of People With Incidentally Detected High Blood Pressure During Health Check‐Up in Korea
Source: Int J Hypertens. 2025 Dec 30;2025:5581168. doi: 10.1155/ijhy/5581168 (PMC12754265; doi:10.1155/ijhy/5581168)
Supplement: Supplementary file 1 — Supporting Information Additional supporting information can be found online in the Supporting Information section. [file IJHY-2025-5581168-s001.docx]

**Supplementary Table 1. Post-hoc Analysis: Demographic data**

| Group 1 | Group 2 | Raw  p-value | adjusted  p-value |
| --- | --- | --- | --- |
| Male (%) | | | |
| Drug-free | Continuous medication | 1.9936E-57 | **1.9936E-56** |
| Drug-free | No re-initiation | 2.3713E-05 | **0.00023713** |
| Drug-free | Re-initiation | 0.00125832 | **0.01258316** |
| Drug-free | The others | 1.0825E-07 | **1.0825E-06** |
| Continuous medication | No re-initiation | 1.983E-12 | **1.983E-11** |
| Continuous medication | Re-initiation | 0.00586455 | 0.05864548 |
| Continuous medication | The others | 1.7676E-05 | **0.00017676** |
| No re-initiation | Re-initiation | 0.26799906 | 1 |
| No re-initiation | The others | 0.11799087 | 1 |
| Re-initiation | The others | 0.96514067 | 1 |
| Current smoking (%) | | | |
| Drug-free | Continuous medication | 4.14753E-41 | **4.14753E-40** |
| Drug-free | No re-initiation | 0.007124403 | 0.071244028 |
| Drug-free | Re-initiation | 0.006060541 | 0.060605407 |
| Drug-free | The others | 4.04896E-10 | **4.04896E-09** |
| Continuous medication | No re-initiation | 6.62364E-14 | **6.62364E-13** |
| Continuous medication | Re-initiation | 0.006154651 | 0.061546513 |
| Continuous medication | The others | 0.010205386 | 0.102053856 |
| No re-initiation | Re-initiation | 0.157519313 | 1 |
| No re-initiation | The others | 0.000454482 | **0.004544818** |
| Re-initiation | The others | 0.35952689 | 1 |
| Diabetes mellitus (%) |  |  |  |
| Drug-free | Continuous medication | 1.53058E-09 | **1.53058E-08** |
| Drug-free | No re-initiation | 2.6327E-05 | **0.00026327** |
| Drug-free | Re-initiation | 0.022449998 | 0.224499978 |
| Drug-free | The others | 2.30773E-14 | **2.30773E-13** |
| Continuous medication | No re-initiation | 0.50830387 | 1 |
| Continuous medication | Re-initiation | 0.897807449 | 1 |
| Continuous medication | The others | 0.017615274 | 0.176152736 |
| No re-initiation | Re-initiation | 0.815154616 | 1 |
| No re-initiation | The others | 0.005645622 | 0.056456221 |
| Re-initiation | The others | 0.14388617 | 1 |
| Dyslipidemia (%) |  |  |  |
| Drug-free | Continuous medication | 1.75504E-08 | **1.75504E-07** |
| Drug-free | No re-initiation | 0.043687124 | 0.436871236 |
| Drug-free | Re-initiation | 0.217770308 | 1 |
| Drug-free | The others | 0.002986328 | **0.029863283** |
| Continuous medication | No re-initiation | 0.051236248 | 0.512362484 |
| Continuous medication | Re-initiation | 0.414310771 | 1 |
| Continuous medication | The others | 0.549630171 | 1 |
| No re-initiation | Re-initiation | 0.807881972 | 1 |
| No re-initiation | The others | 0.312611375 | 1 |
| Re-initiation | The others | 0.683643312 | 1 |
| Systolic blood pressure (mmHg) |  |  |  |
| Continuous medication | Drug-free | -8.2547 | **0** |
| Continuous medication | No re-initiation | -4.1917 | **0** |
| Continuous medication | Re-initiation | -1.0481 | 0.4429 |
| Continuous medication | The others | -0.4456 | 0.81 |
| Drug-free | No re-initiation | 4.063 | **0** |
| Drug-free | Re-initiation | 7.2066 | **0** |
| Drug-free | The others | 7.8092 | **0** |
| No re-initiation | Re-initiation | 3.1436 | **0** |
| No re-initiation | The others | 3.7461 | **0** |
| Re-initiation | The others | 0.6025 | 0.8992 |
| Diastolic blood pressure (mmHg) |  |  |  |
| Continuous medication | Drug-free | -3.4433 | **0** |
| Continuous medication | No re-initiation | -2.0783 | **0** |
| Continuous medication | Re-initiation | -1.2652 | 0.0978 |
| Continuous medication | The others | -0.4115 | 0.7366 |
| Drug-free | No re-initiation | 1.3649 | **0** |
| Drug-free | Re-initiation | 2.178 | **0.0001** |
| Drug-free | The others | 3.0317 | **0** |
| No re-initiation | Re-initiation | 0.8131 | 0.5312 |
| No re-initiation | The others | 1.6668 | **0** |
| Re-initiation | The others | 0.8537 | 0.5383 |
| BMI (kg/m²) |  |  |  |
| Continuous medication | Drug-free | -0.125 | 0.3905 |
| Continuous medication | No re-initiation | -0.1266 | 0.7441 |
| Continuous medication | Re-initiation | -0.0036 | 1 |
| Continuous medication | The others | -0.1914 | 0.5319 |
| Drug-free | No re-initiation | -0.0015 | 1 |
| Drug-free | Re-initiation | 0.1215 | 0.9601 |
| Drug-free | The others | -0.0664 | 0.9708 |
| No re-initiation | Re-initiation | 0.123 | 0.9693 |
| No re-initiation | The others | -0.0649 | 0.9878 |
| Re-initiation | The others | -0.1879 | 0.8902 |

BMI, Body Mass Index.

Note: p-values are Bonferroni / Tukey HSD adjusted. Significant values (p<0.05) are bolded.

**Supplementary Table 2. Post-hoc Analysis: Laboratory data**

| Group 1 | Group 2 | Mean diff | adjusted  p-value |
| --- | --- | --- | --- |
| FBS (mg/dL) | | | |
| Continuous medication | Drug-free | -2.503 | **0.00e+00** |
| Continuous medication | No re-initiation | -1.558 | 0.2531 |
| Continuous medication | Re-initiation | 5.395 | **0.0005** |
| Continuous medication | The others | 0.868 | 0.8755 |
| Drug-free | No re-initiation | 0.945 | 0.5239 |
| Drug-free | Re-initiation | 7.898 | **0.00e+00** |
| Drug-free | The others | 3.372 | **0.0001** |
| No re-initiation | Re-initiation | 6.954 | **0.00e+00** |
| No re-initiation | The others | 2.427 | 0.0851 |
| Re-initiation | The others | -4.527 | **0.0163** |
| Total Cholesterol (mg/dL) | | | |
| Continuous medication | Drug-free | -4.398 | **0.00e+00** |
| Continuous medication | No re-initiation | -1.754 | 0.5141 |
| Continuous medication | Re-initiation | -0.296 | 0.9999 |
| Continuous medication | The others | -1.247 | 0.8792 |
| Drug-free | No re-initiation | 2.644 | **0.0219** |
| Drug-free | Re-initiation | 4.102 | 0.1581 |
| Drug-free | The others | 3.151 | **0.0412** |
| No re-initiation | Re-initiation | 1.458 | 0.9490 |
| No re-initiation | The others | 0.507 | 0.9963 |
| Re-initiation | The others | -0.951 | 0.9915 |

FBS, fasting blood sugar.

Note: p-values are Tukey HSD adjusted (family-wise). Significant values (p<0.05) are bolded.

**Supplementary Table 3. Post-hoc Analysis : The incidence of cardiovascular events**

| Event | Comparison | Raw  p-value | adjusted  p-value |
| --- | --- | --- | --- |
| Angina | Drug-free vs Continuous | **5.79e-18** | **5.79e-17** |
| Angina | Drug-free vs No re-initiation | **1.21e-09** | **1.21e-08** |
| Angina | Drug-free vs Re-initiation | **2.24e-07** | **2.24e-06** |
| Angina | Drug-free vs The others | **6.54e-07** | **6.54e-06** |
| Atrial fibrillation | Drug-free vs N No re-initiation | **4.53e-03** | **4.53e-02** |
| Atrial fibrillation | Drug-free vs Re-initiation | **2.83e-04** | **2.83e-03** |
| Cerebral Infarct | Drug-free vs No re-initiation | **2.05e-04** | **2.05e-03** |
| Cerebral Infarct | Drug-free vs Re-initiation | **5.26e-06** | **5.26e-05** |
| Cerebral Infarct | Drug-free vs The others | **6.61e-06** | **6.61e-05** |
| Cerebral Infarct | Continuous vs Re-initiation | **2.10e-03** | **2.10e-02** |
| Heart failure | Drug-free vs Continuous | **1.81e-15** | **1.81e-14** |
| Heart failure | Drug-free vs No re-initiation | **3.36e-10** | **3.36e-09** |
| Heart failure | Drug-free vs Re-initiation | **6.51e-08** | **6.51e-07** |
| Heart failure | Drug-free vs The others | **1.18e-09** | **1.18e-08** |
| IHD | Drug-free vs Continuous | **2.59e-23** | **2.59e-22** |
| IHD | Drug-free vs No re-initiation | **3.47e-10** | **3.47e-09** |
| IHD | Drug-free vs Re-initiation | **2.45e-08** | **2.45e-07** |
| IHD | Drug-free vs The others | **1.89e-08** | **1.89e-07** |
| CKD | Drug-free vs Continuous | **2.09e-08** | **2.09e-07** |
| CKD | Drug-free vs No re-initiation | **2.11e-04** | **2.11e-03** |
| CKD | Drug-free vs Re-initiation | **8.29e-04** | **8.29e-03** |
| CKD | Drug-free vs The others | **1.12e-09** | **1.12e-08** |
| PAOD | Drug-free vs Continuous | **1.11e-44** | **1.11e-43** |
| PAOD | Drug-free vs No re-initiation | **3.48e-06** | **3.48e-05** |
| PAOD | Drug-free vs Re-initiation | **4.01e-08** | **4.01e-07** |
| PAOD | Drug-free vs The others | **1.00e-04** | **1.00e-03** |
| PAOD | Continuous vs No re-initiation | **5.60e-07** | **5.60e-06** |
| PAOD | Continuous vs The others | **7.24e-05** | **7.24e-04** |

IHD, Ischemic heart disease; CKD, chronic kidney disease; PAOD, peripheral arterial occlusive disease.

Note: p-values are Bonferroni-adjusted. All of the significant results are shown and bolded (p<0.05).
